# Virtually supported penicillin allergy de-labelling during COVID-19

**DOI:** 10.1186/s13223-023-00770-x

**Published:** 2023-02-27

**Authors:** Arian Ghassemian, Geetanjalee Sadi, Raymond Mak, Stephanie Erdle, Tiffany Wong, Samira Jeimy

**Affiliations:** 1 Division of Allergy and Immunology, Department of Medicine, Western University, B3-112, 268 Grosvenor Street, London, ON N6A 4V2 Canada; 2 Division of Allergy and Immunology, Department of Pediatrics, The University of British Columbia, Vancouver, BC Canada; 3 Division of Allergy and Immunology, Department of Medicine, The University of British Columbia, Vancouver, BC Canada

**Keywords:** Drug allergy, Telemedicine, Penicillin allergy, Antimicrobial stewardship, COVID-19

## Abstract

**Background:**

Penicillin allergy is a commonly listed medication allergy despite rare overall incidence. Many patients erroneously have this label, which has personal, health, and societal costs. Penicillin allergy delabelling requires an oral challenge, which can be a rate limiting step in the de-labeling process; this is even more relevant with the reduction of in-person visits during the COVID-19 pandemic.

**Objective:**

To identify the utility and broader applicability of using a virtually supported platform, initially adopted given COVID-19 restrictions, to expedite penicillin oral provocation challenge and penicillin de-labeling in patients at low to moderate risk of immediate hypersensitivity reaction and based on shared decision making.

**Methods:**

Patients in Vancouver catchment area were referred for penicillin allergy and virtually assessed by the consulting allergist between July 2020 and April 2021. Those deemed appropriate for oral challenge based on the allergist consultant were offered the option of a virtual oral provocation challenge to oral amoxicillin in a subsequent virtual visit. Patients who agreed and were consented underwent a virtually supervised oral amoxicillin challenge during the second virtual visit. Findings are summarized in this case series.

**Results:**

Twenty-three patients, both adult and pediatric, ranging from no to significant co-morbidities were consented and underwent the virtual challenge. One hundred percent of patients were successful with no reaction after an hour post virtual oral provocation challenge with amoxicillin.

**Conclusion:**

Virtual medicine is likely to remain in the allergist’s practice. Virtually supported penicillin allergy delabelling, based on shared decision making and risk stratification, presents another pathway for penicillin allergy delabelling.

## Introduction

Penicillin allergy is the most listed medication allergy in the general population [1]. Five to 15% of patients in developed countries carry a penicillin allergy label; however, anaphylaxis is exceedingly rare, occurring in 0.001–0.0005% of patients [1]. A penicillin allergy label leads to use of alternate antimicrobial agents with more side effects, and to increased rates of multidrug resistant and hospital acquired infections [1]. Erroneous labels of penicillin allergy can prolong hospitalizations and lead to higher readmission rates and higher patient care costs in both inpatient and outpatient setting [2].

Given the personal and societal costs, allergy and immunology organizations [3, 4] recommend proactive penicillin allergy de-labelling. However, even before the COVID-19 pandemic, access to penicillin allergy testing was a rate limiting step in the de-labelling process, particularly in Canada. The gold standard for de-labelling is an oral challenge (OC) with the suspected drug [3], with a growing body of evidence supporting direct OC (without skin testing), if the clinical history is appropriate [3, 5–7]. Faced with similarly urgent need for allergy assessment for food allergy and food introduction in the setting of the COVID-19 pandemic, a Canada wide collaboration established a risk stratification model allowing virtually supervised allergenic food introduction to infants at risk of food allergy [8]. Given the success of the at-risk food introduction model and the ongoing COVID pandemic and restrictions at the time, we identified a need and opportunity to evaluate changes in clinical practice that could offer more remote options for allergist intervention, starting with penicillin de-labelling. Penicillin allergy, risk stratification, and OC are well studied with validated tools. If our practice adaptations using these tools were successful, they could become a permanent option in the event of remote access and could outlive the pandemic. The allergists involved in these virtually supported penicillin allergy de-labelling practices thus performed risk stratification (based on a combination of personalized risk/benefit assessment and a published Canadian consensus statement [3]) and virtually supervised low to intermediate risk amoxicillin oral challenges, to establish an alternate pathway for penicillin allergy de-labelling. The collective findings were reviewed leading to the current submission. The objective of this study is to summarize our experience as well as to identify the utility and broader applicability of using a virtually supported platform, initially adopted given COVID-19 restrictions, to expedite penicillin oral provocation challenge and penicillin de-labeling in patients at low to moderate risk of immediate hypersensitivity reaction and based on shared decision making.

## Methods

This case series describes the use of virtually supported platforms that allowed for remote and virtual delabelling of penicillin allergy using a one step amoxicillin OC. With the limitations of the pandemic, starting in July 2020, patients in the Vancouver and surrounding catchment area who were referred for penicillin allergy were assessed virtually by independent allergists practicing in the community and tertiary health care centres in Vancouver, British Columbia. Given adaptation necessitated by the pandemic restrictions, the option for an amoxicillin OC was offered to all patients and families that were deemed suitable based on (1) the initial review of their reactions, (2) cardiovascular reserve and ability to tolerate anaphylaxis, and (3) urgency of penicillin allergy delabelling. The option for virtual challenge was offered on a case-by-case basis depending on the inflow of relevant referrals from the community. Those individuals who were willing to accept the risk were included in the current communication. There was no pre-specified timeline for this clinical endeavour. The following baseline data was collected: patients’ age, sex, comorbidities, inciting medication, index reaction, risk of reaction, rationale for virtually supervised challenge, and subsequent outcome (Table 1).

**Table 1:** Demographic of patient who underwent virtual challenge

Age	Sex	Comorbidities	Inciting medication	Index reaction	Risk of reaction^a^	Rationale for virtually supervised challenge based on patient–physician shared decision making	Reactions—immediate or delayed^b^
3	M	Multiple food allergies	Amoxicillin	Generalized urticaria	Intermediate	Convenience–distance	None
3	F		Amoxicillin	Maculopapular rash	Intermediate	Convenience–distance	None
4	M	Cystic fibrosis	Amoxicillin clavulanic acid	Maculopapular rash	Intermediate	Convenience–distance	None
4	M	Asthma, atopic dermatitis	Amoxicillin	Maculopapular rash	Intermediate	Convenience–distance	None
6	F	Recurrent urinary tract infections, allergic rhinoconjuctivitis	Amoxicillin	Generalized urticaria	Intermediate	Convenience–distance	None
6	F	Atopic dermatitis	Amoxicillin	Maculopapular rash	Intermediate	Convenience–distance	None
6	F		Amoxicillin clavulanic acid	Urticaria	Intermediate	Convenience–distance	None
8	F		Amoxicillin	Generalized urticaria and facial angioedema	Intermediate	Convenience–distance	None
8	M	NSAID allergy	Amoxicillin	Urticaria and angioedema	Intermediate	Convenience–distance	None
8	M	Cystic fibrosis	Amoxicillin	Urticaria	Intermediate	Convenience	None
11	F		Amoxicillin	Generalized urticaria	Intermediate	Convenience	None
12	M	Food allergy (peanut), asthma, allergic rhinoconjunctivitis	Penicillin	Maculopapular rash	Intermediate	Convenience, less missed school days	None
12	F		Amoxicillin	Generalized urticaria	Intermediate	Convenience	None
12	M		Amoxicillin	Maculopapular rash	Intermediate	Convenience	None
14	M	Allergic rhinitis, food allergy, asthma	Amoxicillin	Urticaria	Intermediate	Convenience	None
19	F	Asthma	Amoxicillin	Generalized urticaria	Intermediate	Convenience, less missed school days	None
37	M	Relapsing Hodgkin lymphoma, thyroiditis, previous stem cell transplant	Amoxicillin	Rash	Intermediate	Reduce hospital/infectious risk exposure	None
54	F	Interstitial lung disease on oxygen (end stage)	Amoxicillin	Localized rash on arm	Intermediate	Reduce hospital/infectious risk exposure in patient-high risk for severe COVID19 outcomes	None
65	F	Myelodysplastic syndrome, dyslipidemia, hypertension, diabetes	Piperacillin tazobactam	Petechial macules/thin papules coalescing into larger purpuric patches on her upper and lower extremities and including trunk, patient also thrombocytopenic	Intermediate	Reduce hospital/infectious risk exposure in immunosuppressed patient	None
66	F	Multiple myeloma awaiting stem cell transplant, hypertension, diabetes	Amoxicillin	Local rash to chest	Intermediate		None
70	F	Locally advanced thymic tumour, hypertension, diabetes	Penicillin	Urticaria	Intermediate	Reduce hospital/infectious risk exposure in patient-high risk for severe COVID19 outcomes	None
70	F	Diffuse large B-cell lymphoma awaiting bone marrow transplant	Penicillin	Unknown	Intermediate	Convenience	None
71	M	Interstitial lung disease (end stage) awaiting transplant, emphysema, hypothyroidism, hypertension, dyslipidemia	Penicillin	Local rash	Intermediate	Reduce hospital/infectious risk exposure in patient-high risk for severe COVID19 outcomes	None

All patients underwent a one-step oral challenge with 250–500 mg of oral amoxicillin (or weight-based dose if pediatric) at their physician’s discretion. Majority of patients lived over 2 h from the centre and/or had co-morbidities putting them at high risk of severe COVID, thus wishing for limited healthcare interaction

^a^Risk of reaction for proposed oral challenge determined as per the algorithm recommended in reference 3

^b^Reactions—immediate or delayed after the oral challenge took place

The initial consult was conducted on a secure platform which included a video and audio component as this would allow the physician to obtain a baseline appreciation of patient health status within the limitations of virtual visits. During the initial consultation, history was obtained to determine the characteristics of the index reaction, risk of true allergy and risk of adverse reaction to a provocation challenge, according to the Canadian Society of Allergy and Clinical Immunology (CSACI) position statement on beta lactam allergy [3]. The algorithm in the CSACI Position Statement [3] stratifies patients according to risk of future reaction to beta lactam antibiotics and provides guidance on beta-lactam introduction or oral provocation challenge if patients are at low to intermediate risk of reaction. Based on this algorithm, patients that were deemed appropriate for an amoxicillin OC were stratified to the intermediate risk category that still met the criteria to proceed to direct OC, which corresponds to a score of 0–2 (very low to low risk) on the PEN-FAST clinical decision rule. The option to undergo a virtual challenge along with the risks and benefits of the challenge was presented to all patients deemed to be at low to intermediate risk of reaction as per the CSACI beta-lactam allergy risk stratification algorithm [3]. Twenty-three patients accepted the risk and provided verbal informed consent. Features of anaphylaxis, including when to seek medical attention, were discussed. Patients were explicitly told they were able to reconsider at any time including the date of the challenge. All patients who accepted the risk of a virtual challenge between the period of July 2020 to April 2021 are included in the current communication.

Patients who were eligible and consented to proceed with a virtual amoxicillin OC were prescribed amoxicillin for their challenge. Prescriptions for the appropriate dose was called or faxed to their community pharmacy by the attending physician and patients secured the amoxicillin prescription prior to the day of their OC. On the day of the challenge, patients met with their supervising physician on a secure video platform, and a review of their health status was performed to ensure safety. Verbal consent was reviewed, and a single step oral challenge was then carried out, while the physician was on standby via video for 60 min for immediate assistance and guidance in case of a reaction. Patients were required to have a capable friend, family member, or guardian available in their vicinity to function as a caretaker, and counselling provided on when, how, and where to seek immediate medical attention in the event of an adverse reaction requiring immediate care. Given the index history for each patient, a risk of anaphylaxis was felt to be low enough such that no new epinephrine prescriptions were provided for the purpose of the amoxicillin OC alone. Three pediatric patients, ages 3, 12, and 14 had previously diagnosed food allergies and epinephrine auto-injector prescriptions. Caregivers of pediatric patients were in direct contact with the attending physician.

**Fig. 1:**
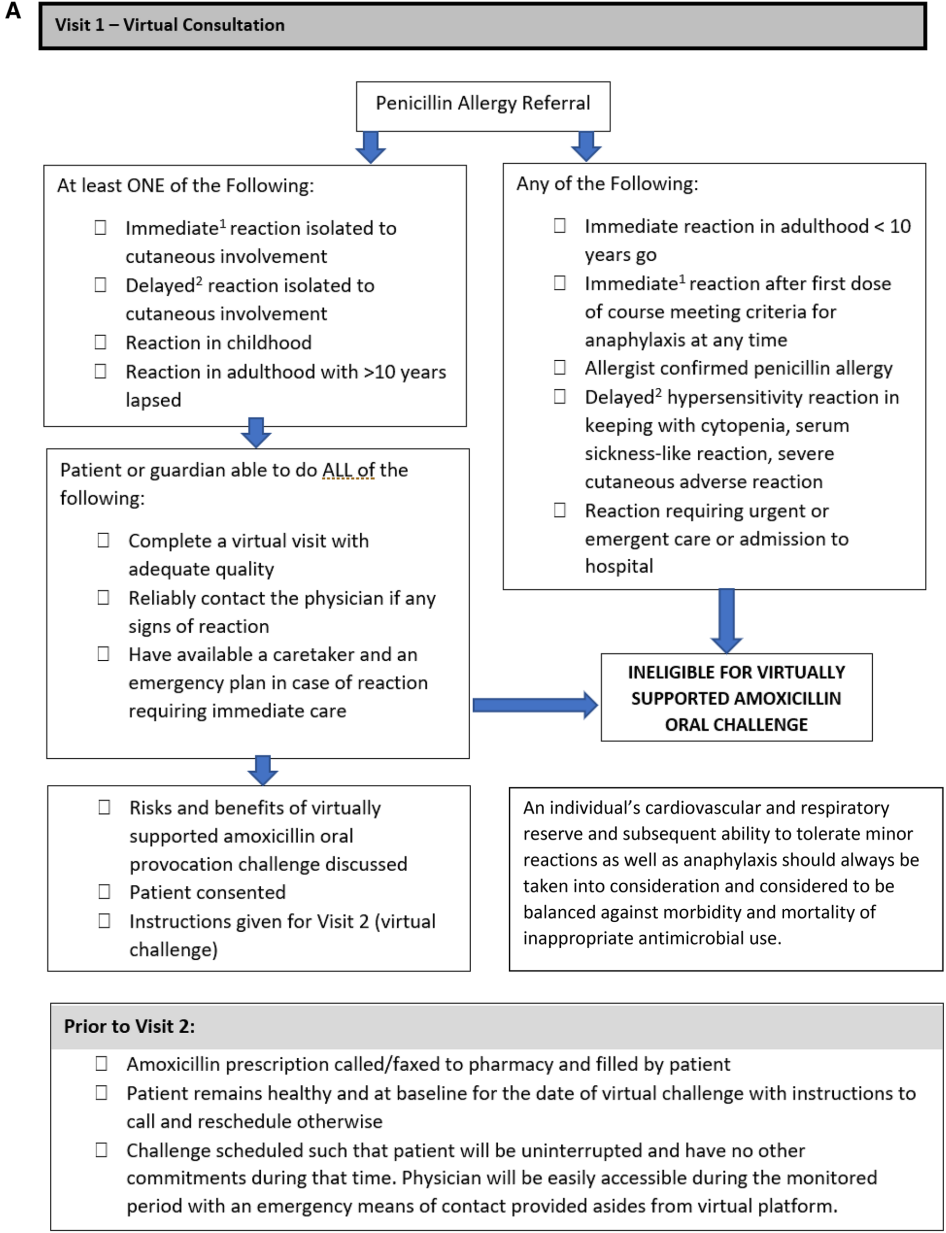

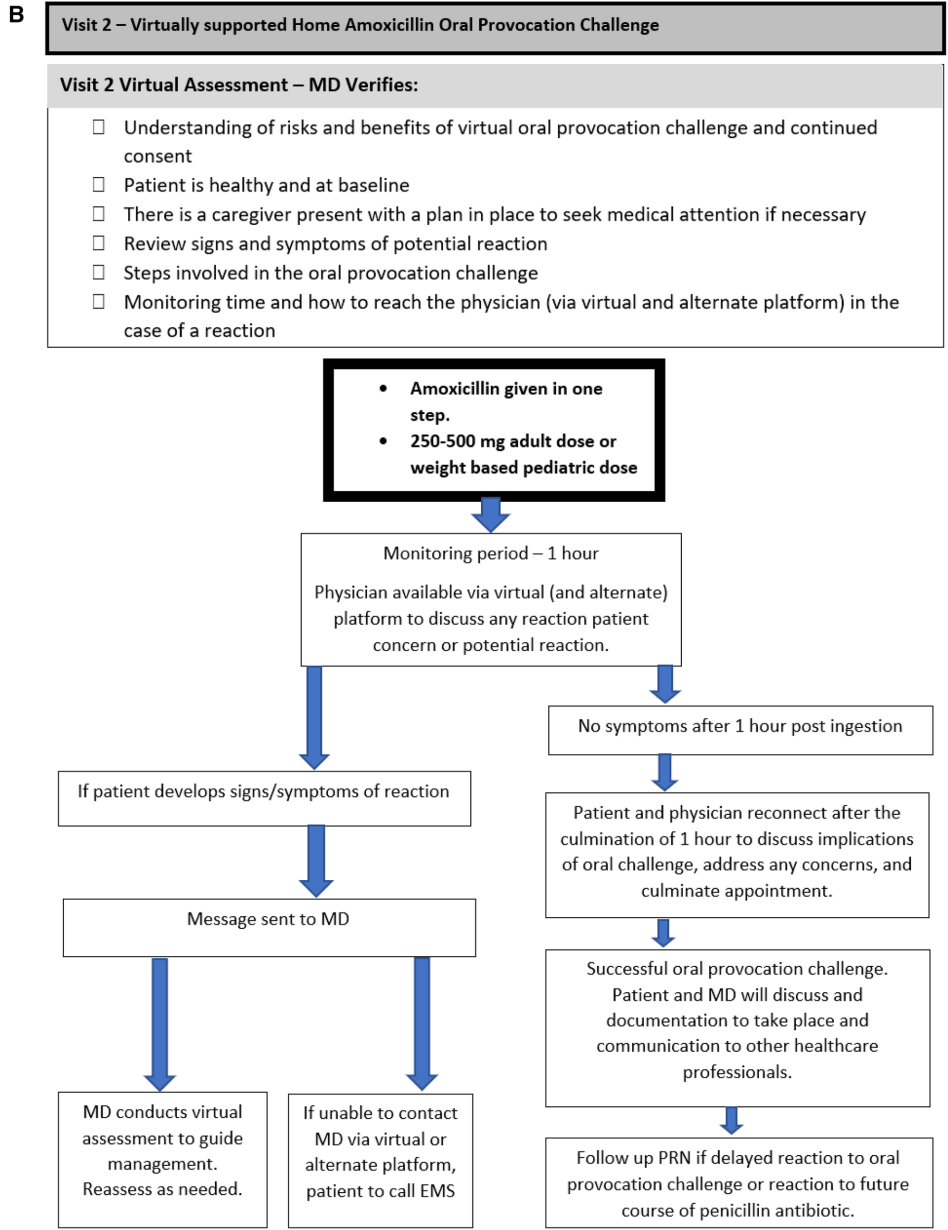
A flow chart and checklist of a virtually supported penicillin oral challenge. **A** Visit 1: virtual consultation and preparation. **B** Visit 2: virtually supported home amoxicillin oral provocation challenge. (1) Immediate reaction is defined as Type I hypersensitivity reaction that is IgE mediated and occurs within 2 h of the first dose of medication and lasts < 24 h. (2) Delayed hypersensitivity reaction are defined as Type II–IV reactions that typically take > 24 h to develop and raise concern for end organ involvement (cytopenias, renal/hepatic dysfunction, serum sickness), or severe cutaneous adverse reactions (skin desquamation, purpura, mucosal lesions, SJS/TEN, DRESS, AGEP) (Figure adapted from Mack et al. [8]. JACI: In Practice)

Two virtual visits were required for the virtual challenge to take place, with the initial virtual visit functioning as consultation and assessment of eligibility for the virtual challenge, and the second virtual visit functioning as the visit where the remote OC took place with the algorithm summarized in Fig. 1. Risks, benefits, and consent to proceed with the virtual amoxicillin OC was discussed during both encounters.

After successful completion of the amoxicillin OC, patients were counselled on the low risk of delayed reactions and when to seek urgent medical care if required. They were provided with instructions to contact the attending physician in the event of any delayed reaction. No formal follow up was scheduled at the end of the OC if patients were otherwise well, with follow up planned on an as needed basis in the event of delayed reactions to the amoxicillin OC or in the context of a course of amoxicillin taken for a subsequent infection.

## Results

Twenty-three patients completed the virtually supervised challenge. Demographic information and reaction characteristics are outlined in Table 1. Clinical history revealed the majority of the patients experienced isolated cutaneous symptoms, including urticaria, non-urticarial exanthem, and mild angioedema. The majority of patients had also experienced mild, delayed cutaneous reactions, occurring > 24 h from onset of treatment. Finally, over half the patients undergoing the amoxicillin OC had their index reaction prior to age 18 and had at least 1 year pass since the reaction. A detailed description of reaction characteristics is outlined in Table 2. In the patients who were offered virtual amoxicillin OC, all previous penicillin reactions were self-limited and resolved within 2–4 days of cessation of antibiotic use with or without the use of antihistamines for symptom relief. Epinephrine was not required for any of the index reactions.

**Table 2:** Reaction history and characteristics of patients who underwent the virtually supervised oral amoxicillin challenge

Reaction history and characteristics	Proportion of patients
Symptoms concerning for cytopenia, serum sickness-like reactions, severe cutaneous adverse reactions	None
Timing to reaction from first dose	
• Immediate (< 6 h)	1 (4.3%)
• Delayed (> 24 h)	16 (69.6)
• Unknown	6 (26.1%)
Cutaneous involvement only^a^	19 (83%)
• Generalized urticaria	7 (30.4%)
• Non-urticarial exanthem	11(47.8%)
• Angioedema	1 (4.3%)
• Remote, unknown	1 (4.3%)
Index reaction prior to age 18	15 (65.2%)
Time elapsed since index reaction	
• > 1 year	21 (91.3%)
• < 1 year	2 (8.7%)

^a^With no history of severe cutaneous adverse reactions

Of the 23 patients challenged, no patients experienced a reaction during the observation period of the virtually supervised challenge. No delayed reactions have been reported to date, either as a consequence of the challenge, or with treatment with a penicillin antibiotic. A minimum of 7 months has elapsed since the last virtual amoxicillin OC reported in this paper.

## Discussion

This diverse group of patients included adults with significant medical comorbidities at baseline and would almost certainly require antibiotics in the near future, including patients awaiting bone marrow and solid organ transplants. Although some of these patients were deemed to have limited cardiovascular reserve, the urgency of delabelling the antibiotic allergy took precedence, based on shared decision making with the allergist. Despite the variable age and health status, with appropriate patient selection, there was a 100% success rate in re-introduction of amoxicillin through a virtually supervised OC.

With the limitations of the COVID-19 pandemic, modifications of ambulatory allergy/immunology services were needed to appropriately triage patients and while we are currently seeing a return to in-person visits, we must learn from our past experiences. Shaker et al. proposed a patient prioritization schematic recommending that except for certain patient populations within the specialty, such as those with primary immunodeficiency, there is limited need for face-to-face visits [9]. Our study is the first demonstration that virtually supported penicillin allergy de-labeling is a reasonable method of health care delivery, including in different age groups, and in the presence of co-morbidities as illustrated in our small sampling. When carried out by an experienced professional, who conducted risk stratification of (1) penicillin anaphylaxis and (2) ability to withstand anaphylaxis, and then mutually agreed on delabelling in appropriately selected patients, our virtually supported penicillin de-labelling has led to positive results, de-labelling patients in a timely and cost effective manner during the pandemic.

An erroneous label of penicillin allergy confers significant burden to patients, and to an already stretched health care system. The process of de-labeling penicillin allergy was made even more challenging given access limitations due to the ongoing COVID-19 pandemic. Penicillin de-labelling allows for safer and more broad-spectrum therapeutic options for the outpatient setting. Virtual care can be central in providing services within a risk-stratified context [9]. The need for social distancing in doctor’s offices, to limit exposure to vulnerable individuals, and for rapid evaluation provided rationale for this new model of penicillin allergy assessment.

Limitations of this adaptive practice may include the one hour of observation after a single step amoxicillin challenge, given that many patients had a history of delayed reactions. A recent study by Van Gasse et al. demonstrated negligible value in prolonged drug challenges in the context of delayed or unclear reactions. In their study, only three of 128 patients undergoing a prolonged challenge had reactions. The reactions were mild maculopapular cutaneous reactions, which alone would not be a contraindication to the drug. In our virtual delabelling protocol, patients were advised to notify their attending physician in the event of any reaction thought to be due to the single step OC or due to a course of a penicillin antibiotic after delabelling. This would allow for recognition of a wider patient population and provide real world data for clinical decision making and antimicrobial guidance and would allow patients to have penicillin antibiotics as an available medication in the interim.

Another limitation in the current communication is the small number of patients described. Given the limitations of patient referrals and suitability for a remote challenge, the small number of patients who agreed to undergo virtually supervised challenge do represent a wide spectrum of ages and health statuses and indicates feasibility for larger-scaled studies. We describe our clinical findings as a prospective first step for future studies as well as a pragmatic means of empowering our colleges with similar challenges faced around penicillin de-labelling. There is both pediatric and adult data reporting the safety of amoxicillin OC without skin prick testing [5, 7]. The heterogenous nature of sampling highlights this fact suggesting that as a treatment option, virtual amoxicillin OC and penicillin de-labelling can have a profound impact across the age spectrum. Patient selection and steps taken in this study closely resemble the practice of many allergists across the country. Yet, we would like to caution that the risk of any individual patient reacting increases with increasing frequency of challenges. As such, we stress the importance of a careful discussion and shared decision making around risk and benefits, as no oral challenge, regardless of index reaction history or personal health status is without risk. For clinicians who may want to adopt virtual penicillin de-labelling option to their practice, steps can be taken to mitigate the potential for adverse outcomes, even if not adopted in the current communication. The risk and benefits of acquiring an epinephrine auto-injector solely for the purpose of the challenge is one such reasonable option and a great juncture for shared decision making. Alternatively, we can recruit the help of our community medicine colleagues to arrange more local yet well-equipped environments for patients at high risk of adverse outcome due to limited cardiovascular or respiratory reserve.

Finally, the results obtained are that of two independent practitioners in Vancouver BC and by its very nature cannot at this time be considered to be broadly generalizable but allows for the dissemination of knowledge and experience that may be applicable in specific settings.

## Conclusion

Virtual amoxicillin OC in the carefully selected patient may be an approach to management that can be adopted long after the restrictions of the pandemic have been lifted and represents a shift in paradigm for drug allergy testing to allow patients to have access to optimal antimicrobial agents when needed, especially those residing in remote locations where travel presents an added layer of complexity to the assessment.

## Abbreviations

CSACI: Canadian Society of Allergy and Clinical Immunology
OC: Oral challenge
SCAR: Severe cutaneous adverse reaction

## References

[1] Blumenthal KG, Peter JG, Trubiano JA, Phillips EJ (2019). Antibiotic allergy. Lancet.

[2] Castells M, Khan DA, Phillips EJ (2019). Penicillin allergy. N Engl J Med.

[3] Jeimy S, Ben-Shoshan M, Abrams EM, Ellis AK, Connors L, Wong T (2020). Practical guide for evaluation and management of beta-lactam allergy: position statement from the Canadian Society of Allergy and Clinical Immunology. Allergy Asthma Clin Immunol.

[4] Lang DM, Castells MC, Khan DA, Macy EM, Murphy AW (2017). Penicillin allergy testing should be performed routinely in patients with self-reported penicillin allergy. J Allergy Clin Immunol Pract.

[5] Mill C, Primeau MN, Medoff E, Lejtenyi C, O’Keefe A, Netchiporouk E, Dery A, Ben-Shoshan M (2016). Assessing the diagnostic properties of a graded oral provocation challenge for the diagnosis of immediate and nonimmediate reactions to amoxicillin in children. JAMA Pediatr.

[6] Trubiano JA, Vogrin S, Chua KY, Bourke J, Yun J, Douglas A, Stone CA, Yu R, Groenendijk L, Holmes NE, Phillips EJ (2020). Development and validation of a penicillin allergy clinical decision rule. JAMA Intern Med.

[7] Tucker MH, Lomas CM, Ramchandar N, Waldram JD (2017). Amoxicillin challenge without penicillin skin testing in evaluation of penicillin allergy in a cohort of Marine recruits. J Allergy Clin Immunol Pract.

[8] Mack DP, Hanna MA, Abrams EM, Wong T, Soller L, Erdle SC, Jeimy S, Protudjer JL, Chan ES (2020). Virtually supported home peanut introduction during COVID-19 for at-risk infants. J Allergy Clin Immunol Pract.

[9] Shaker MS, Oppenheimer J, Grayson M, Stukus D, Hartog N, Hsieh EW, Rider N, Dutmer CM, Vander Leek TK, Kim H, Chan ES (2020). COVID-19: pandemic contingency planning for the allergy and immunology clinic. J Allergy Clin Immunol Pract.

